# Leveraging Xenobiotic-Responsive Cancer Stemness in Cell Line-Based Tumoroids for Evaluating Chemoresistance: A Proof-of-Concept Study on Environmental Susceptibility

**DOI:** 10.3390/ijms252111383

**Published:** 2024-10-23

**Authors:** Ki-Hyung Kim, Seung Joon Lee, Juil Kim, Yuseok Moon

**Affiliations:** 1 Laboratory of Mucosal Exposome and Biomodulation, Department of Integrative Biomedical Sciences, Pusan National University, Yangsan 50612, Republic of Korea; ghkim@pusan.ac.kr (K.-H.K.); fovero1007@gmail.com (S.J.L.); 1022myths@hanmail.net (J.K.); 2 Department of Obstetrics and Gynecology, College of Medicine, Pusan National University, Busan 49241, Republic of Korea; 3 Biomedical Research Institute, Pusan National University, Busan 49241, Republic of Korea; 4 Graduate Program of Genomic Data Sciences, Pusan National University, Yangsan 50612, Republic of Korea

**Keywords:** cancer stemness, spheroid cultures, chemoresistance, ribosome-inactivating stressors, environmental susceptibility

## Abstract

Emerging evidence suggests that cancer stemness plays a crucial role in tumor progression, metastasis, and chemoresistance. Upon exposure to internal or external stress, ribosomes stand sentinel and facilitate diverse biological processes, including oncological responses. In the present study, ribosome-inactivating stress (RIS) was evaluated for its modulation of cancer cell stemness as a pivotal factor of tumor cell reprogramming. Based on the concept of stress-responsive cancer cell stemness, we addressed human intestinal cancer cell line-based off-the-shelf spheroid cultures. Intestinal cancer cell line-based spheroids exhibited heightened levels of CD44^+^CD133^+^ cancer stemness, which was improved by chemical-induced RIS. Further evaluations revealed the potential of these stress-imprinted spheroids as a platform for chemoresistance screening. Compared to adherent cells, stemness-improved spheroid cultures displayed reduced apoptosis in response to 5-fluorouracil (5-FU), a frontline chemotherapeutic agent against colorectal cancer. Moreover, serial subcultures with repeated RIS exposure maintained and even enhanced cancer stemness and chemoresistance patterns. In particular, isolated CD44^+^CD133^+^ cancer stem cells exhibited higher chemoresistance compared to unsorted cells. To elucidate the mechanisms underlying RIS-induced stemness, RNA-seq analysis identified Wnt signaling pathways and stemness-associated signals as notable features in spheroids exposed to RIS. Loss-of-function studies targeting connective tissue growth factor (CTGF), a negative regulator of Wnt signaling, revealed that CTGF-deficient spheroids exhibited improved cancer stemness and resistance to 5-FU, with RIS further enhancing these effects. In conclusion, this proof-of-concept study demonstrates the feasibility of leveraging stress-responsive cancer stemness for the development of spheroid-based platforms for chemoresistance evaluation and elucidation of pathophysiological processes of colorectal tumorigenesis under environmental stress.

## 1. Introduction

Conventional in vitro 2D monolayer culture is insufficient to represent in situ phenotypes and cell–matrix interactions and, thus, fails to recapitulate cellular functions of tissues and human physiology. Although animal models reproduce in vivo physiology closely, it is inadequate for drug screening because of fundamental differences between animals and humans in biology and genetic background. The lack of ensured translatable pre-clinical drug screening models, which hamper the general drug development pipeline but also companion diagnostics, is the main cause of the exceptional elevation of costs and paucity of new drug approvals [1,2]. Due to the advent of next-generation sequencing and the democracy of analytical techniques, various types of tumors can be sequenced, and understanding of their genetic alterations was improved in great detail. However, the efficacy of precise chemotherapy remains difficult to predict before clinical decisions, largely due to the lack of standardized preclinical models that accurately mimic human physiology and drug responses. Considering this limitation, human physiology-based drug screening platforms in vitro have been robustly developed [3,4,5]. Three-dimensional spheroid or organoid-based pharmaceutical screening platforms have emerged and proposed potential alternative methodologies to recapitulate human physiology and genetic settings. Organoids can be cultured in vitro from embryonic stem cells, induced pluripotent stem cells, adult stem cells such as intestinal epithelial stem cells, which reside in the crypt, and even tumor tissues from patients. Organoids are more beneficial and valid than animal and even patient-derived xenograft models in terms of accuracy, specificity, efficacy, and disease modeling. Compared to patient-derived xenografts, organoids can be tested against an unlimited amount of compounds and combinations and established in almost all patients. Tumoroids are in great detail a well-verified genetic representation of tumors, even personalized characters; therefore, organoid-based platforms can facilitate addressing the gap between molecular features and patient outcomes [6,7]. Despite the potent advantage of normal and patient-derived organoid platforms, the establishment of organoid from stem cells, i.e., embryonic, adult, and induced pluripotent stem cells, was a major huddle for the standardization of culture and drug testing, and a lack of resources increased the extraordinary cost. Despite an incomplete model system, two-dimensional cell cultures have been widely used due to many advantages, including simplicity, accessibility to biochemical assessment techniques, the convenience of manipulation, and high reproducibility.

Increasing evidence demonstrates that human cancers, including gut malignancy, are polygenic multifactorial diseases, and environmental factors are crucial modifiers in the disease progression [8]. Repeated occurrences of these abnormal responses and ongoing stress heighten the likelihood of developing serious mucosal disorders, including inflammatory bowel disease, bowel fibrosis, and colorectal cancer [9,10,11]. Moreover, the dysregulation of translation in the metabolism of cancer cells underscores the importance of ribosomes in the development of cancer. Furthermore, the recent identification of somatic mutations in ribosomal proteins across various cancers reinforces the connection between ribosomal anomalies and cancer progression [12]. Upon exposure to internal or external stress, ribosomes stand sentinel and facilitate diverse oncogenic processes in stress-insulted cells and tissues [13,14,15,16,17,18]. In particular, emerging evidence suggests that cancer stemness plays a crucial role in tumor progression, metastasis, and chemoresistance under cellular stress [16,17,18,19]. In the present study, it was hypothesized that cellular stress can modulate cancer stemness as a pivotal factor in reprogramming the tumor spheroids. Moreover, the tumor cell line-derived spheroids or organoids were assumed to be more advantageous than ones from stem cells and tissues for drug development or research purposes in developmental biology and in situ phenotypes. Considering limitless, simplified, and standardized resources for organoid establishment, the cancer cell line-derived spheroids and organoids would provide a reproducible cancer stemness-featuring platform for drug discovery and toxicological evaluations.

## 2. Results

### 2.1. Proof-of-Concept Study for Off-the-Shelf CSCs-Based Spheroid Cultures

**Figure 1:**
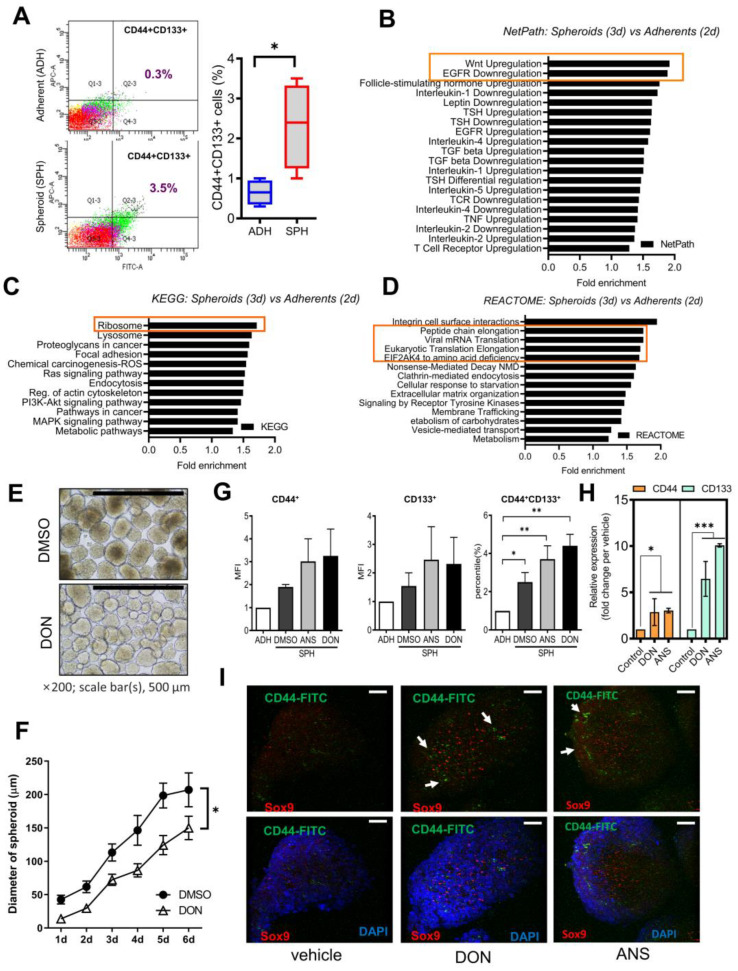
(**A**) Adherent (ADH) or spheroid (SPH) HCT-8 cells were stained for CD44 (FITC) and CD133 (APC) and quantified using the flow cytometer. Dots in a spectrum of colors represent the levels of cell clustering based on localization. The values in the upper right domains indicate levels of double-positive cancer cell population (%). Statistical analysis was conducted using data from four replicates, each consisting of 100 spheroids. Results are shown as a plot with Tukey whiskers, and asterisks (*) indicate significant differences between the two groups (* *p* < 0.05). (**B**) Based on the transcriptome analysis, NetPath analysis of human signaling transduction pathways from the manually curated resource was performed based on differentially expressed genes (DEGs) in spheroids compared with adherent HCT-8 cells. (**C**,**D**) Based on the transcriptome analysis, functional analyses of differentially expressed genes between spheroids (3D) vs. adherent HCT-8 cells (2D) were performed using KEGG (Kyoto Encyclopedia of Genes and Genomes, (**C**)) or REACTOME (**D**), which are initiative bioinformatic database tools of biological functionality and visualization pathways, respectively. Orange-colored squares indicate notable features as mentioned in the result. (**E**–**I**) HCT-8 cells were pre-exposed to 500 ng/mL of DON or 50 ng/mL of ANS, then cultured to form spheres for 6 days ((**E**), magnification, 40×; scale bars(s), 500 μm), and measured for the diameter of the spheroid with culture time (**F**). Asterisks represent statistically significant differences between the two groups based on the Wilcoxon rank sum test (* *p* < 0.05). (**G**) Mean fluorescence intensity (MFI) for CD44 and CD133 levels were measured in four replicates of 100 spheroids using flow cytometry analysis, and asterisks (*) indicate significant differences between the two groups (* *p* < 0.05, ** *p* < 0.01). (**H**) mRNA expression of *CD133* and *CD44* in HCT-8 spheroid cell population exposed to RIS and asterisks (*) indicate significant differences from each vehicle control group (* *p* < 0.05, *** *p* < 0.001). (**I**) Protein expression of CD44 and Sox9 in the HCT-8 spheroids using confocal microscopic Z-stacked images (the original magnification of 400×; scale bar(s), 50 μm). White arrows indicate CD44-positive cell clusters.

HCT-8 cell-derived spheroids were initially constructed as a model for tumoroid formation, exhibiting a higher proportion of CD44^+^CD133^+^ double-positive cancer stem cell markers compared to the adherent control cells (Figure 1A).

Based on the transcriptomic changes in signaling molecules, the spheroid formation caused typical signaling patterns, including upregulation of Wnt- and EGFR-related signaling components, which are well-known signaling modules of cell development and stemness (Figure 1B). In the present study, the gut stress-responsive model was created using HCT-8 cells, which are widely used for inflammation and oncogenesis [17,18,20,21]. We compared human intestine-derived cell lines (HCT-8, HT-29, DLD-1, LOVO, and SW480) for Wnt signaling molecules, including activated beta-catenin, c-Myc, and CTGF (Supplementary Figure S1A). In particular, HCT-8 exhibited relatively high levels of beta-catenin and c-Myc compared to the other cell lines. Further functional prediction using the transcriptomic profiling revealed that the ribosome-associated gene expression was notably altered during the spheroid formation in addition to the attachment and cytoskeleton structure-associated factors (Figure 1C). Moreover, ribosome-associated translational machinery and its stress responses are mainly implicated during the process of the spheroid formation (Figure 1D). We thus assessed cytoskeletal structure-related factors involved in tumorigenesis, including epithelial–mesenchymal transition (EMT) molecules, in response to chemical-induced ribosomal stress. In particular, HCT-8 and SW480 cells showed relatively sensitive responses of epithelial–mesenchymal transition to the ribosomal stress (Supplementary Figure S1B). Moreover, the ileocecum of the small intestine, the source of the HCT-8 cell line, is one of the most susceptible regions to ribosomal stress responses in the gastrointestinal tract [22,23]. Therefore, the HCT-8 cell line was chosen as a promising model for environmental stress-responsive transformation during Wnt-linked tumorigenesis. Moreover, the ribosome stalling and collisions are well known to trigger the integrated stress responses, which mediate diverse cellular processes during the patho-logical states [24,25,26]. It was thus assumed that the ribosome-inactivating stressors (RIS), including deoxynivalenol (DON) and anisomycin (ANS), may modulate the cancer cell stemness signaling in the present study. Although exposure to RIS decreased the growth rate and the size of the spheroid compared to the vehicle-treated control (Figure 1E,F), levels of CD44^+^CD133^+^ cancer stemness tended to be improved by the chemical stressors (Figure 1G). Despite a marginal increasing e of each cancer stemness marker at passage 1, levels of the double-positive cell population were notably elevated by RIS (Figure 1G,H). Moreover, levels of Sox9, a key transcription factor in the stem cell zone of the small intestine and colon, were also elevated by DON and ANS exposure in the spheroid (Figure 1I). However, Sox9-positive cells did not exhibit increased levels of the cancer stemness marker CD44. Thereby, the ribosomal stress-enhanced cancer stemness of the present spheroid model was used for securing a cancer cell population from cancer cell lines.

### 2.2. Chemoresistance Evaluation of RIS-Induced Cancer Cell Spheroids

**Figure 2:**
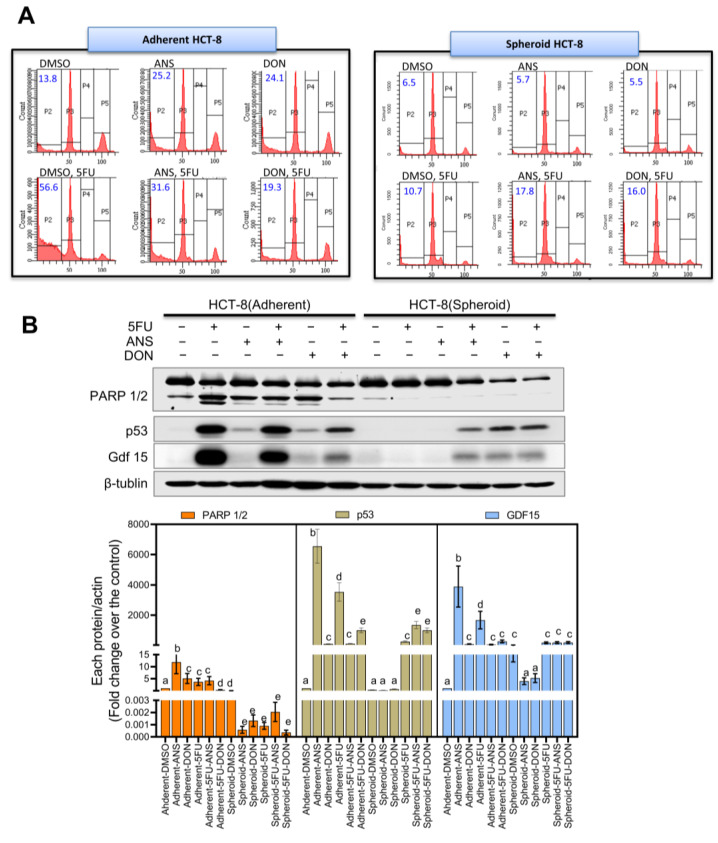
(**A**) Cell cycle analysis of adherent or spheroid HCT-8 cells as measured using propidium iodide (PI) by flow cytometry. Blue values indicate levels (%) of sub-Go population. (**B**) Adherent or spheroid cells were exposed to 500 ng/mL of DON or 50 ng/mL of ANS for 24 h and then treated with 5-FU (375 μM) for 48 h. The cell lysates were subjected to Western blot analysis. Different letters over each box represent statistically significant differences among groups based on one-way ANOVA (*p* < 0.05).

Considering a potent CSC-based platform for chemoresistance screening, the culture system was evaluated for its responses to 5-fluorouracil (5-FU), a frontline chemotherapeutic agent against CRC. Compared to the adherent control cells (56.6%), apoptotic cells and apoptosis markers (PARP1/2, p53, Gdf15 (a target gene of p53) were notably reduced in the spheroid cultured cells (DMSO, 10.7%; DON, 17.8%; ANS, 16.0%) (Figure 2A,B, respectively).

**Figure 3:**
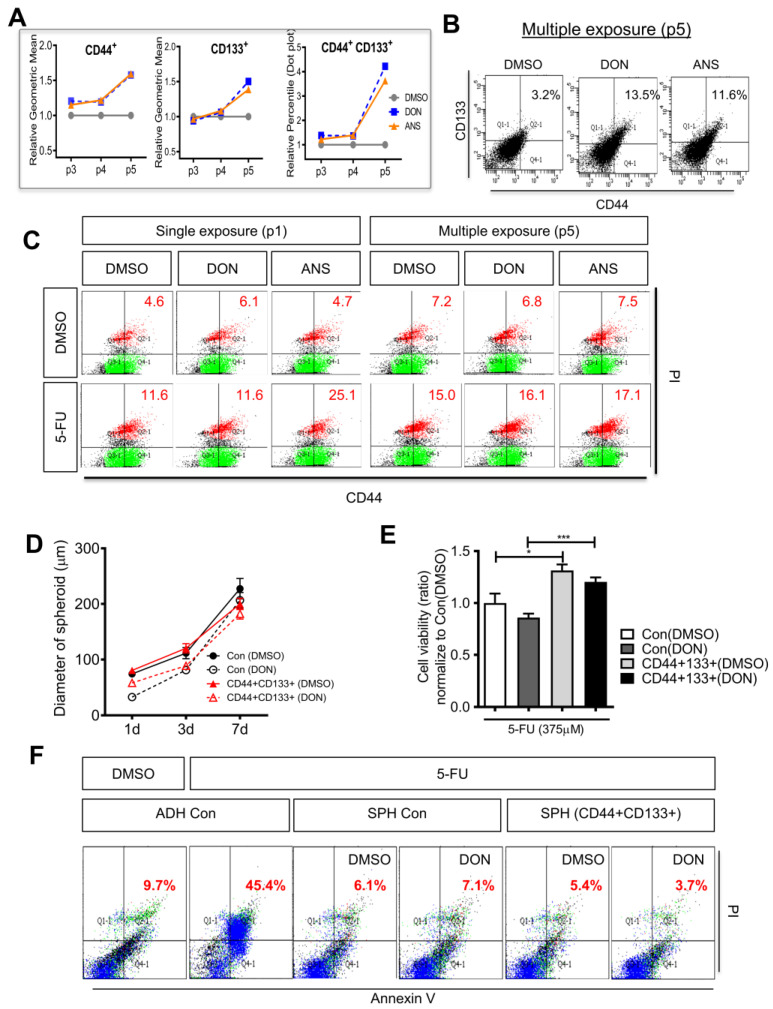
(**A**) Cancer cell spheroids of each passage (a record of the number of times the culture has been sub-cultured after trypsinization) were measured for levels of CD44 and CD133 using flow cytometry assay. In each passage, cells were pre-exposed to 500 ng/mL DON or 50 ng/mL ANS for 24 h and further cultured for 6 days before subculture as indicated in the method. (**B**) Multiply RIS-exposed spheroids (passage 5, p5) were quantified for CD44 and CD133. (**C**) Spheroids single (p1) or multiply (p5) exposed to RIS (500 ng/mL DON or 50 ng/mL ANS) were treated with 5-FU (375 μM) for 48 h, and then measured for CD44 and Propidium Iodide (PI). Red values indicate the levels of CD44^+^ cells in the G2 phase. (**D**) Control and CD44^+^CD133^+^ spheroids were exposed to RIS (500 ng/mL DON) and then measured for the diameter with time. (**E**,**F**) Control and CD44^+^CD133^+^ spheroids were exposed to RIS (500 ng/mL DON) and then treated with 5-FU (375 μM) for 48 h. Cells were measured for their cell viability (**E**) and apoptosis (**F**). Asterisks represent statistically significant values (* *p* < 0.05, *** *p* < 0.001). The 5-FU treatment dose (375 μM) is a level required to inhibit the growth of adherent HCT-8 cells by approximately 50% while the spheroids display resistance. Red values indicate the early apoptosis levels as shown in the upper right domain. Dots in a spectrum of colors represent the levels of cell clustering based on localization.

To reproducibly secure cell line-based cancer stem cells and develop them as a tumoroid platform for chemoresistance evaluation, we investigated whether cancer stemness is maintained during the subculture with repeated RIS exposure. The cancer cell population with RIS-induced stemness displayed a gradual increment with serial subcultures (Figure 3A). In particular, at passage 5, the CD44 and CD133 double-positive cell populations were 4.2-fold and 3.6-fold, respectively (Figure 3B). Moreover, CD44 cancer stem cells at G2 phase levels were increased in response to 5-FU treatment, indicating cells undergo proper DNA maintenance before mitosis in response to genotoxic chemotherapeutic stress (Figure 3C). It was also maintained during the repeated RIS exposure (Figure 3C).

Next, isolated cancer stem cells were verified for their chemoresistance. The CD44^+^CD133^+^ colon cancer stem cell population was sorted, pre-exposed to RIS, and allowed to form spheroids, which were further assessed for their responses to chemotherapeutic 5-FU. Although CD44^+^CD133^+^ colorectal cancer stem cell-based spheroids did not exhibit notably increased size compared with the unsorted cell-based spheroids (Figure 3D), the cell viability response to 5-FU was higher in CD44^+^CD133^+^ sorted spheroid cells than in control spheroid cells (Figure 3E). Consistent with cell viability, 5-FU-induced apoptosis levels were lower in CD44^+^CD133^+^ sorted spheroid cells than in unsorted control spheroid cells, clearly verifying chemoresistance (Figure 3F). In particular, RIS-exposed CD44^+^CD133^+^ sorted spheroid cells displayed more resistance to 5-FU than the unsorted spheroid cells. Taken together, these results suggest that a spheroid-based cultured cell system under RIS facilitates reproducible and easy production of CSCs as a key component of function in an in vitro platform for chemoresistance and other pathophysiological analysis of malignancy-associated features.

### 2.3. Loss-of-Function-Based Evaluation of RIS-Induced Cancer Cell Stemness and Chemoresistance

**Figure 4:**
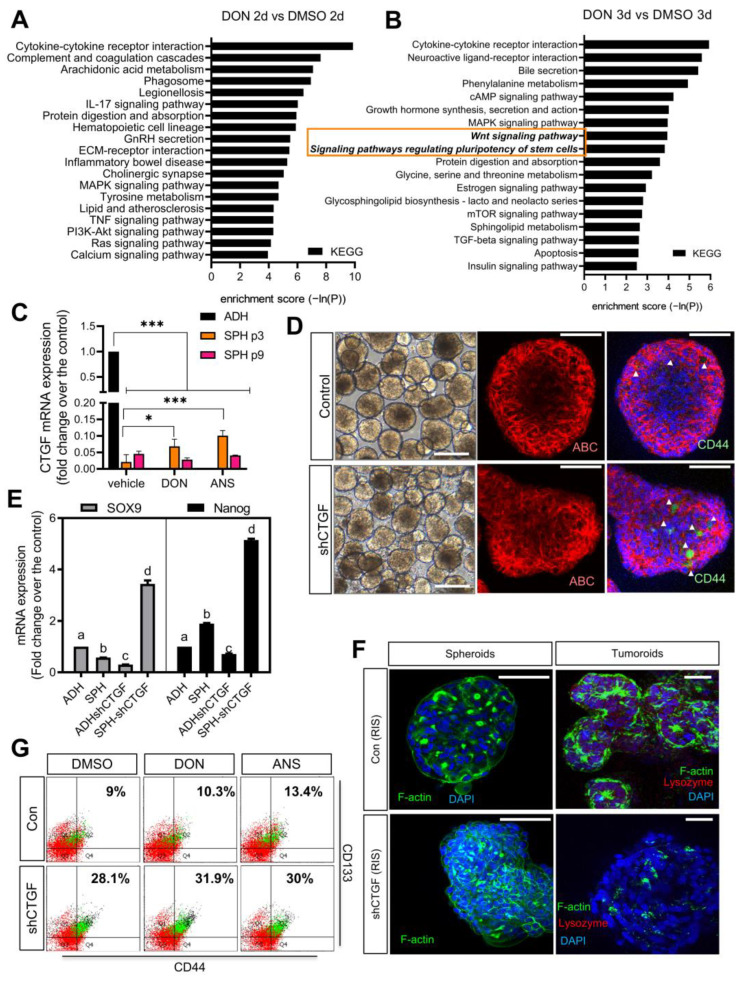
(**A**,**B**) KEGG analysis of DEGs in adherent HCT-8 cells (2D, (**A**)) and spheroids (3D, (**B**)) in response to RIS (DON) exposure. The orange-colored square indicates Wnt and stemness features, as described in the text. (**C**–**E**) Control and CTGF-deficient HCT-8 spheroids were measured for the morphology ((**C**), the original magnification of 40×; scale bar(s), 100 μm), protein expressions (**D**) of CD44, and activated beta-catenin (ABC) using the confocal microscopic analysis (the original magnification of 200×; scale bar(s), 100 μm), and mRNA expression (**E**). Arrows indicate CD44 cells in the spheroids (**D**). (**F**,**G**) Control and CTGF-deficient HCT-8 tumoroids were pre-exposed to RIS (500 ng/mL DON or 50 ng/mL ANS) and measured for CD44 and CD133. Quantitation was performed using the flow cytometry assay (**G**). Dots in a spectrum of colors represent the levels of cell clustering based on localization. The values in the upper right domain indicate levels of double-positive cancer cell population (%). The immune-stained HCT-8 cells were analyzed using confocal microscopy (the original magnification of 200×; scale bar(s), 100 μm, and 400×; scale bar(s), 50 μm for spheroids and tumoroids, respectively). Asterisks represent statistically significant values (* *p* < 0.05, *** *p* < 0.001). Different letters over each box represent statistically significant differences among groups based on one-way ANOVA (*p* < 0.05).

To predict the mechanisms of RIS-improved cancer cell stemness, mRNAs of the intestinal cancer spheroid and adherent cells were evaluated using RNA-seq analysis. Based on the DEG (differentially expressed genes), most DON-induced functional signals were associated with cytokine and pro-inflammatory mediators, particularly in the two-dimensional adherent cancer cells, since DON is well known as an immunotoxic xenobiotic agent leading to acute toxic leucopenia and cytokine dysregulation in lymphocytes and epithelial cells (Figure 4A). However, three-dimensional spheroid cells showed the additional features of stemness-linked signals were enhanced by DON exposure. Wnt signaling pathways and other stemness signaling pathways were notably associated with DON exposure in the HCT-8-based cancer spheroids (Figure 4B). It is consistent with the signaling patterns of spheroids exhibiting remarkable levels of Wnt-related signaling components compared with adherent cells (Figure 1B).

The present platform was further evaluated to address the pathophysiological process through loss of function of Wnt signaling. We created a spheroid platform that is deficient in connective tissue growth factor (CTGF)/cellular communication network factor 2 (CCN2), a critical negative regulator of Wnt signaling, which is closely related to the onset and progression of CRC and regulates cancer stemness by mediating inflammatory response and modulation of epithelial-to-mesenchymal transition (EMT) (2). We reported that the Wnt-CTGF axis in ribosomal insult-associated gastrointestinal oncogenesis was verified by clinical transcriptome profiles [17]. With the attenuation of CTGF signaling, the cancer stemness was improved during the carcinogenesis. CTGF represents a negative regulator of stemness-linked Wnt/β-catenin signaling while it is involved in facilitating the non-conventional Wnt signaling pathways in response to ribosomal inactivation [17]. However, cancer cell line-derived spheroids displayed a remarkable reduction in CTGF compared to the adherent cells, although RIS-exposed spheroids maintained low CTGF expression even by passage 3 (Figure 4C). We further assessed CTGF-deficient cancer cells to address the roles of CTGF in the present model. Although CTGF-deficient spheroids displayed marginal changes in morphology (Figure 4D), stem cell competence was improved by CTGF deficiency. In particular, CD44^+^ colorectal cancer stemness was notably increased by CTGF deficiency (Figure 4D). Since Wnt/β-catenin is upstream signaling of CTGF, levels of activated beta-catenin (ABC) were marginally affected by CTGF deficiency (Figure 4D). However, cellular levels of other stemness markers such as Nanog and Sox9 were notably elevated in CTGF-knockdown spheroids, compared with levels in the control group (Figure 4E). HCT-8 cancer cell line-based spheroid culture was further evaluated in the relatively solid extracellular matrix using matrigel, which supplies essential tumor micronutrients and structural support for the suspended cells. Approximately 37% of the spheroids extended their growth and exhibited bulky and organoid-like morphology. In particular, HCT-8 cell line-derived tumoroids exhibited gut epithelial features such as F-actin lining (Figure 4F). However, CTGF-deficient tumoroids were round and compact, lacking gut organoid-typical cryptic structure. Most CTGF-deficient tumoroids lost the F-actin lining on the surface. We further examined whether RIS triggering affects CTGF-deficiency-mediated colorectal cancer stemness in the tumoroids. RIS partially enhanced CD44^+^CD133^+^ cancer stemness, which was remarkably upregulated by CTGF deficiency (Figure 4G), indicating that CTGF is a crucial regulator of cancer cell stemness.

**Figure 5:**
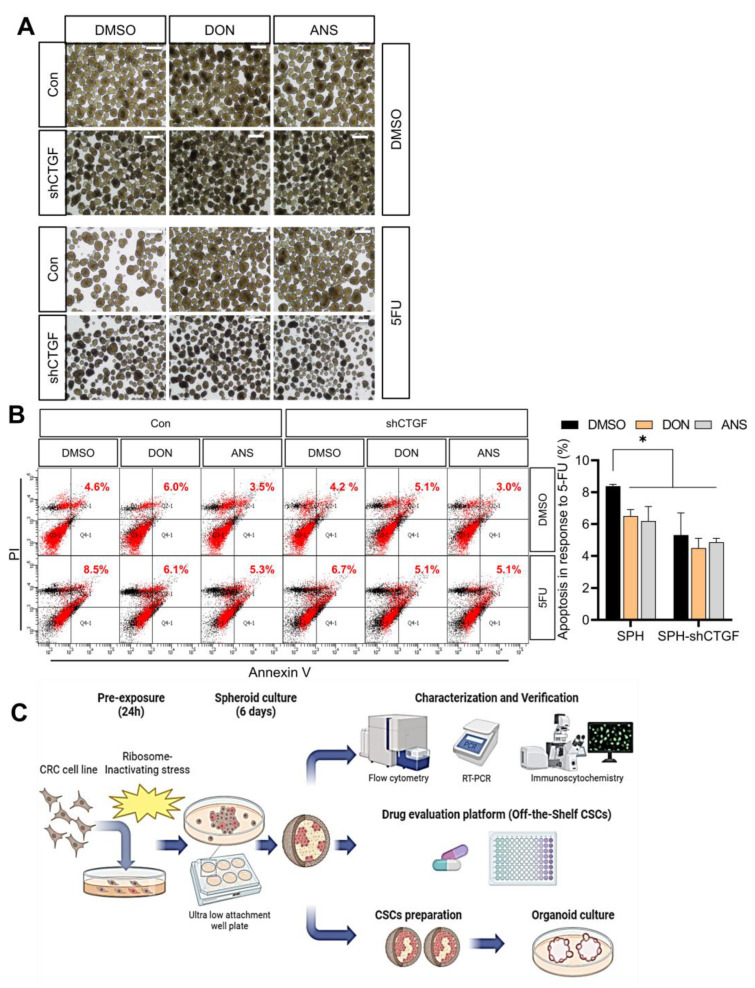
(**A**,**B**) Control and CTGF-deficient spheroids were pre-exposed to RIS (500 ng/mL DON or 50 ng/mL ANS) and then treated with 5-FU (375 μM) for 48 h. Tumor sphere formation (**B**) was observed. The microscopic analysis was performed at the original magnification of 40×; scale bar(s), 100 mm. Moreover, the apoptosis of spheroid cells (**B**) was measured using propidium iodide (PI) and annexin V in response to 5-FU. Red values indicate the early apoptosis levels in each treated spheroids in two replicates of 100 spheroids using flow cytometry analysis, and asterisks (*) indicate significant differences between the two groups (* *p* < 0.05). Dots in a spectrum of colors represent the levels of cell clustering based on localization. (**C**) Schematic overview of the off-the-shelf CSC platform of the anticancer drug efficacy and chemoresistance based on stress-induced cell reprogramming.

Next, the stemness-associated features such as the chemoresistance were assessed in the present model. Although HCT-8 intestinal cancer spheroids exhibited a notable reduction in response to the chemotherapy drug 5-FU, RIS-exposed spheroids demonstrated increased cancer stemness (Figure 4) and resistance to 5-FU-induced toxicity (Figure 5A). Moreover, CTGF-deficient spheroids showed resistance to 5-FU despite morphological shrinkage of the sphere (Figure 5A). As mentioned in Figure 3, approximately half of adherent HCT-8 cells are susceptible to apoptotic cell death when exposed to 375 µM 5-FU. In contrast, the spheroids exhibited resistance to the anticancer agent, with RIS exposure further increasing their resistance to 5-FU-induced apoptosis (Figure 5B). Additionally, CTGF deficiency also contributed to enhanced resistance to 5-FU-induced apoptotic cell death. Taken together, ribosomal stress improves the cancer cell stemness, which contributes to the spheroid formation and the chemoresistance platform using a three-dimensional culture of a cancer cell line. Based on omics-based expression profiling of the chemical-induced model, we predicted biological reprogramming of the tissue and further verified using loss-of-function studies (Figure 5C). Instead of patient-derived cells or tissues, human cancer cell line-based tissue mimics can be used as a potent off-the-shelf platform for proving anticancer drug efficacy and chemoresistance considering the cell stress responses in the real tumor niche.

## 3. Discussion

These spheroids exhibited heightened levels of cancer stemness markers and maintained chemoresistance patterns, offering a promising platform for evaluating anticancer drug efficacy. By employing ribosome-inactivating stressors (RIS), cancer stemness-associated features and chemoresistance were improved in cancer cell line-based tumoroids, resulting in the development of an off-the-shelf screening platform. Furthermore, loss-of-function studies targeting key regulators of Wnt signaling, such as connective tissue growth factor (CTGF), verified the outcomes from RIS-induced stemness and its implications for CRC therapy. In particular, RIS-induced stemness was associated with cancer cell survival in the present model. RIS exposure attenuated chemotherapeutic stress-induced apoptosis responses, which is potently linked to RIS-promoted cancer stemness. Increasing evidence indicates that cancer stem cells, a subset of the cancer cell population, play a key role in chemoresistance and cancer recurrence due to their capacity for self-renewal and differentiation into diverse cancer cell lineages in response to chemotherapy [27,28,29].

Despite the feasible availability of the present platform compared with patient-derived cancer stem cells, this model is limited in its potential for personalized applications. To improve the out-of-shelf assay for customized use, the platform should incorporate patient-derived determinants such as clinical or bioinformatic data. From minimally invasive analysis of the patient tissues, including liquid biopsies, cancer typing and status can be predicted, which can constitute the cell line-derived platform. Based on the ribosomal dysregulation-induced signaling pathway in vitro and clinical data [17], we verified the model using cells deficient in CTGF, a key network regulator of intestinal cancer stemness. Advanced information on genetic determinants from cancer typing would be included to create a cancer subtype-specific platform for chemotherapy decisions.

Instead of cell line-based methods, patient biopsy-derived cancer stem cells are another option for personalized screening platforms such as ChemoID assays to assess the efficacy of chemotherapy and radiotherapy [30,31]. Although chemoID assay-guided therapy improves the prognosis of patients with specific cancer types, such as recurrent glioblastoma, it is limited to patients with operable cancers or healthy conditions. We improved cancer cell stemness, which was chemically maintained during the preparation. Moreover, the present model represents an environmental stress-responsive platform since the ribosome is a crucial sentinel of internal and external insults during chronic distress, including colorectal inflammation and tumorigenesis [18,32,33]. In particular, DON is a most prevalent mycotoxin produced by *Fusarium* fungi that contaminate global staple crops, ultimately posing a harmful risk to animal and human health [34]. Anisomycin has been effectively employed in clinical practice for the treatment of amoebic dysentery and trichomoniasis, and it serves as a crucial active component in Agricultural Antibiotic 120, which is extensively utilized for the management of crop diseases [35]. Mechanistically, DON inhibits protein synthesis by binding to the ribosome and inducing cleavage of the 28S rRNA, which disrupts ribosomal function. Similarly, anisomycin inhibits protein synthesis by interfering with the peptidyl transferase reaction in ribosomes, both of which halt the elongation phase of translation [13,36,37]. In particular, both animals and humans can be frequently exposed to ribosome-targeting agents, such as DON and ANS. Moreover, since most etiologies of the CRC are sporadic, mostly linked to lifestyle and environmental factors, the environmental stress-considered model would recapitulate the real disease process. However, since the stress responses are much more complicated and dynamic, an integrated sensor-containing model would be warranted in future investigations.

Stress-induced cancer stemness represents a paradigm shift in cancer research. Stress stimuli from the microenvironment, including oxidative stress and serum deprivation, are known to upregulate stem-like properties and chemoresistance via activation of Sp1, a stress-responsive transcription factor [38]. Moreover, low levels of oxidative stress increase OCT4 and NANOG, the stemness-associated key transcription factors in head and neck cancers [38]. In response to various types of stressors, insulted cells exhibit dysfunctions of the mRNA translation in the ribosome and subsequent activation of integrated stress responses [12]. Through the activation of stress response pathways, such as the unfolded protein response (UPR) and the integrated stress response (ISR), cells can acquire stem-like properties. This is exemplified by the upregulation of cancer stemness markers and the maintenance of chemoresistance in spheroid cultures exposed to RIS in the present study. Moreover, RNA-seq analysis has identified key signaling pathways, including Wnt signaling, associated with RIS-induced stemness, providing mechanistic insights into this phenomenon. Specifically, ribosomal sentinel-associated proteins play a crucial role in stem cell differentiation and the regulation of both oncogenic and tumor-suppressor genes [12,39]. RIS initiates early remodeling of cell signaling pathways, resulting in changes to intrinsic cancer competence, even though RIS itself does not directly promote tumor cell proliferation [16,17,18]. Investigating the molecular events within the Wnt-CTGF signaling axis will enhance our understanding of how RIS influences cancer cell stemness during colorectal cancer (CRC) progression, offering new insights for potential interventions against CRC.

Another modulation of stress-responsive stemness can be associated with epigenetic regulation in response to stressors, including xenobiotic and inflammatory insults. In particular, the ribosomal stress agents are well-known triggers of inflammatory responses via the production of proinflammatory cytokines [40,41,42,43,44]. Numerous cell types can maintain inflammatory memory, which is potently associated with an epigenetic recording of inflammation. Even after the inflammatory or stressful events have subsided, chromatin remained accessible, and the memory domains displayed distinct histone modifications [45,46]. Memory domains are typically defined as chromatin regions that become accessible during an inflammatory response and remain accessible after the inflammation resolves. Stress-responsive transcription factors such as STAT, C/EBPβ, and AP1 family (including Fos/Jun) are activated during the initial inflammatory response or other stress conditions and are essential for opening memory domains. Loss of these transcription factors led to decreased chromatin accessibility in the memory domains. In particular, histone modifications such as H3K4me1 and H3K27ac are introduced during the initial inflammatory response and often persist at memory domains, indicating a maintained, primed state even after the transcriptional activity of most related genes diminishes [45,46]. One recent study demonstrated that epithelial exposure to RIS such as DON can enhance the enrichment of H3K4me3 via a notable upregulation of histone methyltransferase [47]. Moreover, different types of early stress-responsive transcription factors, including AP1 and early growth response gene 1 product (EGR1), are remarkably elevated by RIS exposure in lymphocytes, macrophages, and epithelial cells [41,42,44,48,49,50]. In addition to the histone modification, stress-responsive EGR1 can regulate DNA methylation during development and diverse stress conditions [51,52]. Moreover, in terms of upstream signaling of the transcription factors, the mitogen-activated protein kinase (MAPK) pathways are remarkably elevated by RIS exposure, mediating the transcription factor activation and subsequent gene expression [41,42,43,44]. Furthermore, persistent low-level activation of the MAPK pathway is a characteristic associated with memory signaling posing an oncogenic potential during tumorigenesis [17,53]. Therefore, stress-responsive MAPK signaling activation and subsequent early responsive transcription factors are predicted to contribute to the epigenetic memory formation in the stress states, including inflammation and tumorigenesis.

A plethora of molecules involved in the central cancer stemness network, including Wnt signaling, are regulated by MAPKs and stress-responsive transcription factors [17]. Functional inactivation of the ribosome leads to the induction of CTGF, a key regulator of Wnt signaling, and proinflammatory chemokine expression, mimicking the early inflammatory responses observed during curative or reparative inflammation in oncogenesis [17]. The eIF2α-mediated integrated stress response (ISR) is the primary mechanism for maintaining cellar homeostasis during endoplasmic reticulum and ribosomal stress. While acute ISR can cause a rapid loss of intestinal epithelial stemness through activation of the unfolded protein response [54], a mild activation of ISR triggers stress sensors that play crucial roles in oncogenic reprogramming and the regulation of cancer stem cell properties [55]. ER stress was shown to counteract the progression of Adenomatous polyposis coli (Apc)-mutated and Wnt hyperactive intestinal stem cells (ISCs) potently through the facilitation of cell differentiation and subsequent repopulation by non-mutated ISCs [56]. In contrast, accumulated adaptation responses during mild ISR can improve cellular survival by triggering NRF2, a master regulator of antioxidants, and metabolic reprogramming as the cancer stemness features [57]. Mechanistically, the NRF2 activation can promote the metabolic reprogramming of cancer cells by enhancing the transcription of genes encoding glycolytic enzymes while inhibiting the conversion of pyruvate to acetyl-CoA by directly activating pyruvate dehydrogenase kinase 1 (PDK1), leading to the inhibition of the tricarboxylic acid (TCA) cycle in the cancer-initiating cells.

In addition to the conventional β-catenin-dependent Wnt pathway, planar cell polarity (PCP) and Wnt/Ca^2+^ signaling pathways are alternately activated depending on the stress regimes. Information about agonists and antagonists of Wnt and other related signaling pathways has expanded our understanding of the crosstalk and mechanisms for a fine-tuning regulation between Wnt/β-catenin and Wnt/PCP signaling in response to RIS. In particular, CTGF represents a convergence point between Wnt/β-catenin and Wnt/PCP signaling pathways in intestinal cancer cells [17]. CTGF counteracts Wnt/β-catenin signaling, which is crucial for maintaining cancer stemness and the growth of colon spheroidal cells [17,58,59]. It is consistent with our result of increased CD44-positive cancer stem cell populations in CTGF-deficient spheroids due to activation of Wnt/β-catenin signaling. At the molecular level, CTGF can interact with the extracellular domain of LRP6, thereby negatively regulating LRP6-mediated Wnt activation. Moreover, Wnt/PCP signaling is essential for CTGF expression and acquiring cancer stemness in response to ribosomal inactivation [17]. Furthermore, cancer progression phase-dependent regulation of CTGF is crucial to the malignancy of latent competent cancer stem cells through the non-canonical Wnt activation, even though the growth of ribosome-insulted cancer cells is decreased. In the present model, cancer cell stemness was remarkably maintained despite growth retardation and morphological shrinkage of the cancer spheroids, indicating a latent action during carcinogenesis. Although latent cancer cells stay in a quiescent state and maintain tumor-initiating capabilities, they can evade the immune system and develop aggressive metastatic traits [17,60]. Stress-related malignant reprogramming in the Wnt-CTGF axis offers new insights into phase-dependent simultaneous monitoring of early cancer cell transformation and latency of relapse in the real tumor niche.

In addition to RIS-induced regulation of cancer cell stemness, RIS exposure is known to regulate the human cell cycle, which can contribute to the chemoresistance of the cancer cells [61,62,63]. Cell cycle arrest can enable cancer cells to avoid the cytotoxic effects of chemotherapy. When cancer cells are in specific phases of the cell cycle, particularly G1 or G2/M phases, they may become less susceptible to drugs designed to target dividing cells. This state of arrest can prevent the incorporation of chemotherapeutic agents into DNA, thereby undermining the treatment’s efficacy [64]. Moreover, alterations in the expression of the cell cycle regulatory proteins, such as cyclins (cyclin D3 and cyclin A) and cyclin-dependent kinases (CDK2), can also contribute to arrest and, consequently, resistance to drugs including 5-FU [64]. In particular, DON enhances cancer cell levels of p21, a central transcriptional regulator of cell cycle arrest via transcriptional, post-transcriptional, or epigenetic regulation [62,63]. However, severe arrest of the cell cycle can lead to a cell death process. High levels of DON can trigger cell death responses while DON-induced p21 counteracts the chemoresistance of cancer cells.

Taken together, these results support the development of a drug screening platform as a promising off-the-shelf cancer stem cell preparation by CSC-based spheroid and organoid culture. The development of spheroid cultures as a platform for chemoresistance evaluation holds great promise for personalized cancer therapy. By recapitulating the three-dimensional architecture and microenvironment of tumor tissues, spheroids and organoids provide a more physiologically relevant model for drug screening or chemoresistance. Notably, RIS-induced cancer stemness enhances the chemoresistance of spheroid cultures, underscoring the importance of considering cancer stemness in drug development efforts. Furthermore, serial subcultures with repeated RIS exposure maintain and even enhance cancer stemness, highlighting the reproducibility and robustness of this approach. The implications of leveraging chemical stress to enhance cancer stemness extend beyond basic research to clinical applications in CRC therapy. By targeting key regulators of Wnt signaling, such as CTGF, it may be possible to modulate cancer stemness and overcome chemoresistance in CRC. Additionally, the development of patient-specific spheroid cultures offers the potential for personalized treatment strategies tailored to individual tumor characteristics. However, challenges remain in translating these findings into clinical practice, including the need for rigorous validation and optimization of experimental protocols. In conclusion, the concept of leveraging chemical stress to enhance cancer stemness represents a promising approach for improving chemoresistance in CRC therapy. Spheroid-based cultures provide a valuable platform for evaluating anticancer drug efficacy and elucidating the mechanisms underlying RIS-induced stemness. Future research efforts should focus on further elucidating the molecular pathways involved, optimizing experimental protocols, and translating these findings into clinical practice to improve patient outcomes in CRC therapy.

## 4. Materials and Methods

### 4.1. Spheroid and Organoid Culture

HCT-8 cells (Catalog #10244, Korean Cell Line Bank, Seoul, Republic of Korea) (2.5 × 10^5^) were seeded in ultra-low attachment 6-well plates (Costar #3471) with cancer stem cell medium (CSCM), which is composed of DMEM/F12 medium supplemented with B27 (Invitrogen, Carlsbad, CA, USA), 20 ng/mL epidermal growth factor (EGF) (BD Science, San Jose, CA, USA), 20 ng/mL basic fibroblast growth factor, 50 units/mL penicillin, and 50 μg/mL streptomycin in a 5% CO_2_ humidified atmosphere at 37 °C. Cells were treated with 500 ng/mL of deoxynivalenol (DON) or 50 ng/mL of anisomycin (ANS) for 24 h for pre-exposure and washed with DMEM/F12 twice, then cultured for 6 days with CSCM.

### 4.2. Flow Cytometry

Spheroid cultured cells were dissociated into single cells with trypsin, washed with PBS, and incubated with FITC-conjugated CD44 (BD Science) and APC-conjugated CD133 (Miltenyi Biotec, Bergisch Gladhach, Germany) antibodies for 15 min at 4 °C. CD44 and CD133 expression was then assessed using a Becton Dickinson FACS Canto II (BD Bioscience, San Jose, CA, USA).

### 4.3. CRC Organoid Culture Using CRC Cell Line-Derived Spheroid

HCT-8 cells were pre-exposed to 500 ng/mL of DON or 50 ng/mL of ANS for 24 h, washed with DMEM/F12 twice, and then cultured for 6 days in the CSCM. Two hundred to four hundred spheroids with approximately 100 μm diameters were homogeneously suspended at 50 μL of CSCM and seeded into individual wells of the 24-well tissue culture plate with 50 μL of chilled matrigel (BD #354324). Spheroids embedded with matrigel were cultured in IntestiCult™ Organoid Growth Medium (Stem Cell Technologies, Vencouver, BC, Canada #06011 and #06012) containing 10 μM Y-27632 for 12 days. The histological and molecular characterization of cultured CRC organoids were examined by whole-mount immunostaining for CD44, E-cadherin, F-actin, and mucin-2.

### 4.4. Plasmid Construction and Transfection

CMV-driven short hairpin RNAs (shRNAs) were constructed by inserting a shRNA into pSilencer 4.1-CMV-neo vector (Ambion, Austin, TX, USA). Vectors containing negative control shRNAs or shRNAs targeting either CTGF are labeled Con or shCTGF, respectively. The negative control shRNA template sequence lacks significant homology to the mouse, human, and rat genome databases. The pSilencer 4.1-CMV neo vector containing the negative control shRNA template was provided by Ambion. Sequences for shRNAs targeting CTGF were 5′-CAC CAG CAT GAA GAC ATA C-3′, respectively. For expression of shRNA, cells were transfected using jetPRIME transfection reagent (Polyplus-transfection, New York, NY, USA), and stably expressing cells were selected according to the manufacturer’s instructions.

### 4.5. RNA-Seq Analysis

Total RNA was extracted using the RiboEX reagent (GeneAll Biotechnology, Seoul, Republic of Korea) by following the protocol provided by the manufacturer. To remove any contaminating DNA, the extracted RNA was treated with DNase. Subsequently, cDNA was synthesized and amplified, and the resulting cDNA library was subjected to high-throughput sequencing using the NovaSeq 6000 platform (Illuumina, San Diego, CA, USA). The quality of the raw sequencing reads was assessed with FastQC. Following quality assessment, reads were cleaned by trimming adapter sequences and removing low-quality sequences with Trimmomatic software v.0.36. Differentially expressed genes (DEGs) were identified under the criteria of a fold change of ≥2 and a raw *p*-value of <0.05 in at least one comparison group. Functional annotation and gene ontology enrichment analysis were conducted using the DAVID Bioinformatics Resources (https://david.ncifcrf.gov/, assessed on 30 December 2023).

### 4.6. Quantitate PCR

RNA was extracted following the manufacturer’s protocol using RiboEX (GeneAll Biotechnology, Seoul, Republic of Korea). Subsequently, 100 ng of RNA from each sample was reverse transcribed into cDNA utilizing Prime Moloney murine leukemia virus reverse transcriptase (Genetbio, Nonsan, Republic of Korea). The amplification process was conducted with n-Taq DNA polymerase (Enzynomics, Seoul, Republic of Korea) on a MyCycler thermal cycler (Bio-Rad, Hercules, CA, USA). The thermal cycling conditions were set as follows: initial denaturation at 95 °C for 2 min, followed by cycles consisting of 30 s of denaturation at 95 °C, 30 s of annealing at 58 °C, and 30 s of elongation at 72 °C. A portion of each PCR product was analyzed through 1% (*w*/*v*) agarose gel electrophoresis and visualized using ethidium bromide (ETBR) staining. The forward and reverse complement PCR primers were *human GAPDH*, 5′-TCA ACG GAT TTG GTC GTA TT-3′ and 5′-CTG TGG TCA TGA GTC CTT CC-3′; *human CD44*, 5′-AGT CAC AGA CCT GCC CAA TG-3′ and 5′-AGC AGG GAT TCT GTC TGT GC3′; *human CD133*, 5′-TGC TGC TTG TGG AAT AGA CAG AAT G-3′, and 5′-AGG AAG GAC TCG TTG CTG GTG AA-3′; *human SOX9*, 5′-GGA AGT CGG TGA AGA ACG GG-3′, and 5′-GAT GTT GGA GAT GAC GTC GC′; and human Nanog, 5′-CTG CAG AGA AGA GTG TCG CA-3′ and 5′-AAA GGC TGG GGT AGG TAG GT-3′. For real-time PCR, FAM was employed as the fluorescent reporter dye, attached to the 5′ ends of the probes to detect amplified cDNA using an iCycler thermal cycler (Bio-Rad, Hercules, CA, USA). The thermal cycling conditions were as follows: an initial denaturation at 94 °C for 2 min, followed by 40 cycles of denaturation at 98 °C for 10 s, annealing at 59 °C for 30 s, and elongation at 72 °C for 45 s. Each sample was tested in triplicate. The relative quantification of gene expression was determined using the comparative threshold cycle (CT) method. The CT value represents the cycle number at which a statistically significant increase in fluorescence is detected. The number of PCR cycles (CT) required for the FAM intensity to surpass a threshold just above the background was calculated for both test and reference reactions. *GAPDH* served as the endogenous control in all experiments.

### 4.7. Western Blot Analysis

Protein expression levels were analyzed using Western immunoblotting. Fifty micrograms of protein were separated by Mini Gel Electrophoresis (Bio-Rad) and transferred onto a PVDF membrane (Pall Corporation, New York, NY, USA). The membranes were then incubated for 2 h at room temperature with the following antibodies: mouse monoclonal anti-human beta-tubulin antibody (1:1000), rabbit polyclonal anti-human PARP 1/2 antibody (1:1000), mouse monoclonal anti-human p53 antibody (1:1000), and mouse monoclonal anti-human GDF15 antibody (1:1000), all obtained from Santa Cruz Biotechnology (Santa Cruz, CA, USA).

### 4.8. Statistical Analysis

Statistical analyses were conducted using GraphPad Prism version 8.01 (La Jolla, CA, USA). For comparing the two groups, Student’s *t*-test was used. For comparisons involving multiple groups, data were analyzed using analysis of variance (ANOVA) with the Newman–Keuls method as the post hoc assessment. Pearson’s correlation analysis was employed to determine the correlation coefficient (R) between two genes.

## Data Availability

Data contained within the article.

## Supplementary Materials

The following supporting information can be downloaded at: https://www.mdpi.com/article/10.3390/ijms252111383/s1.
