# Supplementary material for: Leveraging Xenobiotic-Responsive Cancer Stemness in Cell Line-Based Tumoroids for Evaluating Chemoresistance: A Proof-of-Concept Study on Environmental Susceptibility
Source: Int J Mol Sci. 2024 Oct 23;25(21):11383. doi: 10.3390/ijms252111383 (PMC11545740; doi:10.3390/ijms252111383)
Supplement: Supplementary file 1 [file ijms-25-11383-s001.zip › ijms-3173567-supplementary.pdf]

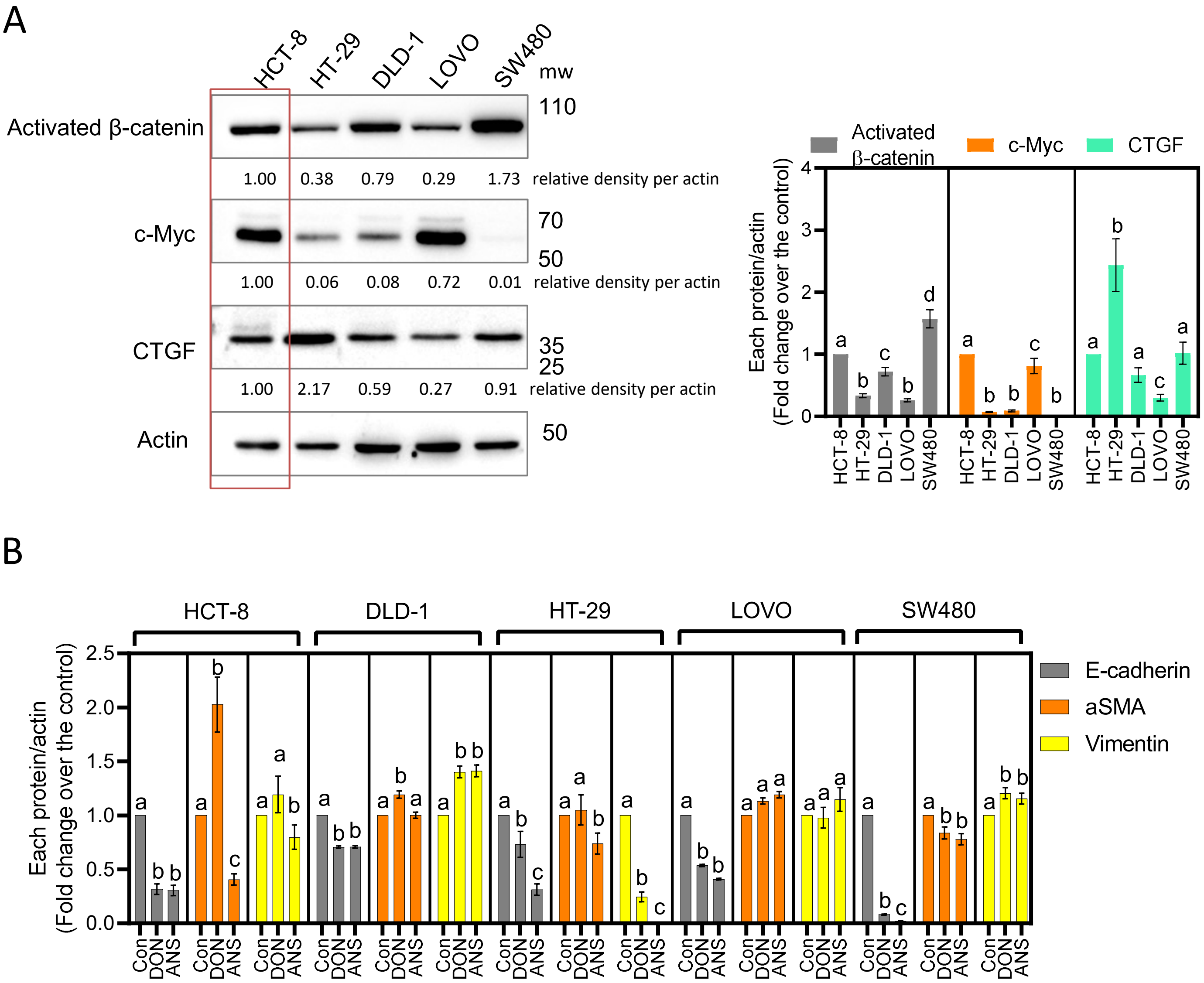

Supplementary Figure S1. (A) Comparison of Wnt signaling protein levels in human intestinal cell lines. (B) human intestinal cells were exposed to 500 ng/mL of DON or 50 ng/mL of ANS for 48 h. All cell lysates were subjected to western blot analysis. Different letters over each box represent statistically significant differences among groups based on one-way ANOVA ($p < 0.05$).
